# Inflammatory biomarker panels in peripheral blood: association with myasthenia gravis onset and severity

**DOI:** 10.3389/fneur.2026.1673022

**Published:** 2026-02-03

**Authors:** Hong Jin, Yuxin Cui, Yunya Ren, Xinmiao Ma, Yishi Wang, Qi Fan, Yulan Cao, Chun-feng Liu, Jing Chen

**Affiliations:** Department of Neurology and Clinical Research Center of Neurological Disease, The Second Affiliated Hospital of Soochow University, Suzhou, Jiangsu, China

**Keywords:** inflammatory biomarkers, myasthenia gravis, myasthenic crisis, neutrophil-to-lymphocyte ratio, risk stratification

## Abstract

**Objective:**

To investigate the association between peripheral blood inflammatory biomarkers and the clinical phenotypes, severity, and prognosis of myasthenia gravis (MG).

**Methods:**

This retrospective study analyzed 134 MG patients (including 23 with myasthenic crisis [MC]) and 58 age- and sex-matched healthy controls hospitalized at the Second Affiliated Hospital of Soochow University (August 2016–March 2024). Peripheral blood inflammatory markers were compared across subgroups. Infection was strictly excluded based on clinical and laboratory criteria. Multivariate logistic regression and receiver operating characteristic (ROC) curve analyses were performed to identify risk factors and diagnostic value.

**Results:**

Compared to controls, MG patients exhibited significantly elevated neutrophil-to-lymphocyte ratio (NLR), platelet-to-lymphocyte ratio (PLR), systemic immune-inflammation index (SII), and systemic inflammation response index (SIRI) (all *p* < 0.05). Patients with MC were characterized by a higher prevalence of generalized MG (GMG) and thymoma, as well as elevated leukocyte counts, NLR, and SIRI compared to non-MC patients. Multivariate analysis identified elevated PLR [OR: 1.01, 95% CI: 1.00–1.02] as independent risk factors associated with MG onset, while elevated NLR [OR: 1.20, 95% CI: 1.05–1.41] and the presence of thymoma [OR: 13.44, 95% CI: 4.42–48.54] were independently associated with MC. Furthermore, inflammatory indices (NLR, PLR, and SII) were significantly higher in GMG and moderate-to-severe cases (MGFA III–V) compared to ocular and mild cases.

**Conclusion:**

Systemic inflammatory biomarkers, particularly PLR and NLR, are significantly elevated in MG and correlate with disease severity and clinical subtypes. While PLR is associated with MG onset, NLR and thymoma are potential indicators for myasthenic crisis. These readily available markers may facilitate risk stratification in clinical practice.

## Introduction

Myasthenia gravis (MG) is a heterogeneous autoimmune disorder of the neuromuscular junction (NMJ) driven by autoantibodies targeting postsynaptic components, primarily the acetylcholine receptor (AChR) (1). Despite standard stratification by age, antibody status, and thymic pathology, predicting the clinical course—particularly the risk of life-threatening myasthenic crisis (MC)—remains challenging (2). MC affects 15–20% of patients, typically within the first few years of diagnosis, and carries a mortality rate of 5–12% (2). While triggers such as infection and surgery are well-recognized, reliable biomarkers to preemptively identify patients at high risk of exacerbation are lacking (3).

Chronic inflammation is central to MG pathogenesis. The dysregulated immune response involves complex interactions between T cells, B cells, and pro-inflammatory cytokines, which ultimately drive autoantibody production (4, 5). Consequently, there is growing interest in peripheral blood inflammatory composites—such as the neutrophil-to-lymphocyte ratio (NLR), platelet-to-lymphocyte ratio (PLR), systemic immune-inflammation index (SII), and systemic inflammation response index (SIRI)—as accessible surrogates for systemic immune activation (6). These markers have demonstrated prognostic utility in various autoimmune and inflammatory conditions (7–9). However, data regarding their specific role in MG remains limited and inconsistent. Previous studies have yielded conflicting results regarding the predictive value of these indices for disease onset versus severity. This study aims to clarify the clinical utility of a comprehensive panel of inflammatory biomarkers (NLR, PLR, LMR, SII, and SIRI) in characterizing MG subtypes and assessing the risk of myasthenic crisis in a Chinese cohort.

## Methods

### Study population

We retrospectively screened 134 MG patients admitted to the Department of Neurology at the Second Affiliated Hospital of Soochow University between August 2016 and March 2024. Diagnosis was confirmed based on typical fluctuating muscle weakness combined with positive serology (anti-AChR antibodies), electrophysiological evidence, or neostigmine testing. Fifty-eight age- and sex-matched healthy individuals served as controls.

### Exclusion criteria

To minimize confounding, we applied strict exclusion criteria: (1) Age <18 or >80 years; (2) Coexisting autoimmune or neuroinflammatory disorders; (3) Active infection (defined by fever, radiological evidence, or CRP > 10 mg/L unrelated to MG); (4) Malignancy; (5) Severe hepatic/renal dysfunction; and (6) Acute cerebrovascular events.

### Data collection

Demographic and clinical data, including Myasthenia Gravis Foundation of America (MGFA) classification (10), Quantitative Myasthenia Gravis Score (QMGS), and thymic imaging, were retrieved from electronic medical records (11). Patients were stratified by age of onset into early-onset MG (EOMG, onset age <50 years) and late-onset MG (LOMG, onset age ≥50 years) (12). Disease severity was dichotomized into mild (MGFA I–II) and moderate-to-severe (MGFA III–V) (13).

### Laboratory analysis

Fasting venous blood samples were collected within 24 h of admission. Complete blood counts and biochemical profiles were analyzed using standard automated analyzers (BC-6800, Mindray; Olympus Au5400). Inflammatory indices were calculated as follows:

- NLR: Neutrophil count/Lymphocyte count
- PLR: Platelet count/Lymphocyte count
- LMR: Lymphocyte count/Monocyte count
- SII: (Platelet × Neutrophil)/Lymphocyte
- SIRI: (Monocyte × Neutrophil)/Lymphocyte

The standard laboratory reference intervals in our institution are as follows: white blood cells (3.5–9.5 × 10^9/L), neutrophils (1.8–6.3 × 10^9/L), lymphocytes (1.1–3.2 × 10^9/L), monocytes (0.1–0.6 × 10^9/L), platelets (125–350 × 10^9/L), and CRP (<3.0 mg/L). Based on these parameters, the normal reference ranges for the composite inflammatory indices are considered to be: NLR (1.0–3.0), PLR (90–250), LMR (2.21–9.89), SII (180–600), and SIRI (<1.0) (7).

### Statistical analysis

Data normality was assessed using the Shapiro–Wilk test. Continuous variables exhibiting a normal distribution were expressed as mean ± standard deviation (SD) and compared using Student’s *t*-test. Conversely, variables with a skewed distribution were presented as median (interquartile range, IQR) and analyzed via the Mann–Whitney *U* test. Categorical variables were expressed as frequencies (percentages) and analyzed using the Chi-square test or Fisher’s exact test. Least Absolute Shrinkage and Selection Operator (LASSO) regression was utilized to screen for significant predictors to minimize multicollinearity. Subsequently, multivariate logistic regression models determined independent risk factors for MG onset and MC, adjusting for potential confounders. Receiver operating characteristic (ROC) curves were generated to evaluate diagnostic performance. A *p*-value <0.05 was considered statistically significant. To control for type I error inflation due to multiple comparisons among inflammatory markers, the Bonferroni correction was applied.

## Results

### Comparison of general clinical data between the MG group and the control group

A total of 192 participants were enrolled in this study, comprising 134 patients with MG and 58 healthy controls. The MG cohort included 64 males (47.8%) and 70 females (52.2%). Regarding treatment history prior to admission, 28 patients (20.9%) had received corticosteroid therapy, and 16 (11.9%) had undergone immunosuppressive treatment. Clinical subtyping revealed that 59 cases (44.0%) were early-onset MG (EOMG), while 75 cases (56.0%) were late-onset MG (LOMG). Ocular MG (OMG) was identified in 33 patients (24.6%), whereas the majority presented with generalized MG (GMG) (*n* = 101, 75.4%). Co-occurring thymoma was found in 36 patients (26.9%). According to the MGFA classification, 59 patients (44.0%) had mild disease (Class I, II), and 75 (56.0%) had moderate-to-severe disease (Class III–V). The control group consisted of 27 males (46.6%) and 31 females (53.4%). There were no statistically significant differences in age or sex distribution between the two groups (*p* > 0.05). Regarding the inflammatory profile, initial analysis suggested differences in multiple markers. However, after adjusting for multiple comparisons to minimize Type I errors, the MG group exhibited significantly higher levels of neutrophil count (*P*-adjust = 0.035), NLR (*P*-adjust = 0.007), PLR (*P*-adjust = 0.007), and SII (*P*-adjust <0.001) compared to controls1. Conversely, although white blood cell count, lymphocyte count, LMR, and SIRI showed nominal differences in the unadjusted analysis, these differences were not statistically significant after adjustment (*P*-adjust > 0.05) (Table 1).

**Table 1:** Comparison of general clinical data between the MG group and the control group.

**Characteristics**	**MG (*n* = 134)**	**Control (*n* = 58)**	***P-value***	***P-adjust***
Age (years old)	53.00 (40.25,67.75)	52.50 (44.75,62.75)	0.911	1.000
Sex (Male, %)	64, 47.8%	27, 46.6%	1.000	1.000
Hormone (%)				
Y	28, 20.9%			
N	106, 79.1%			
Immunosuppressor(%)				
Y	16, 11.9%			
N	118, 88.1%			
Onset time (%)				
EOMG (<50)	59, 44.0%			
LOMG (≥50)	75, 56.0%			
Clinical classification (%)				
OMG	33, 24.6%			
GMG	101, 75.4%			
Thymoma (%)				
Y	36, 26.9%			
N	98, 73.1%			
MGFA (%)
Mild (I, II)	59, 44.0%			
Moderate-to-Severe (III, IV, V)	75, 56.0%			
QMGS	11.69±4.94			
Leucocyte(10^9/L)	6.30 (5.00,8.50)	6.00 (4.82,7.10)	0.042*	0.763
Thrombocyte (10^9/L)	214.00 (178.00,257.75)	203.50 (179.00,251.00)	0.343	1.000
Lymphocyte (10^9/L)	1.60 (1.00.2.20)	1.90 (1.42,2.30)	0.016*	0.287
Neutrophil (10^9/L)	3.90 (3.10,6.10)	3.55 (2.82,4.00)	0.002**	0.035*
Monocyte (10^9/L)	0.40 (0.30,0.50)	0.40 (0.30,0.50)	0.957	1.000
CRP (mg/L)	5.30 (5.00,5.80)	5.35 (5.00,5.60)	0.657	1.000
HDL (mmol/L)	1.27 (1.14,1.47)	1.22 (1.00,1.40)	0.049*	0.874
NLR	2.33 (1.72,4.49)	1.72 (1.41,2.43)	<0.001***	0.007**
MHR	0.29 (0.19,0.40)	0.31 (0.21,0.43)	0.288	1.000
LMR	4.33 (3.36,5.94)	5.00 (4.07,6.34)	0.021*	0.380
PLR	135.17 (107.83,205.00)	113.29 (88.93,139.61)	<0.001***	0.007
SIRI (10^9/L)	0.86 (0.52,1.60)	0.69 (0.48,0.94)	0.005**	0.092
SII (10^9/L)	535.43 (366.49,1027.46)	372.48 (267.75,547.88)	<0.001***	<0.001***

MG, myasthenia gravis; EOMG, early-onset myasthenia gravis; LOMG, late-onset myasthenia gravis; OMG, ocular myasthenia gravis; GMG, generalized myasthenia gravis; MGFA, Myasthenia Gravis Foundation of America; QMGS, quantitative myasthenia gravis score; CRP, C-reactive protein; HDL, high-density lipoprotein; NLR, neutrophil-to-lymphocyte ratio; MHR, monocyte-to-HDL ratio; LMR, lymphocyte-to-monocyte ratio; PLR, platelet-to-lymphocyte ratio; SIRI, systemic inflammation response index; SII, systemic immune inflammation index.

*p < 0.05, **p < 0.01, ***p < 0.001.

### Comparison of general clinical data between the MC group and the non-MC group

Following the comparison with healthy controls, we further stratified the 134 MG patients into a myasthenic crisis (MC) group (*n* = 23) and a non-MC group (*n* = 111) to identify risk factors associated with crisis events.

The two subgroups were comparable in terms of demographic characteristics, with no statistically significant differences observed in age (*P*-adjust = 1.000) or sex distribution (*P*-adjust = 1.000). Similarly, treatment history prior to admission (corticosteroids or immunosuppressants) and the age of disease onset (EOMG vs. LOMG) did not differ significantly between the groups (*P*-adjust > 0.05).

Regarding clinical phenotypes, the presence of thymoma was identified as a robust discriminator between the groups. The prevalence of thymoma was markedly higher in the MC group (69.6%) compared to the non-MC group (18.0%), a difference that remained highly significant after adjusting for multiple comparisons (*P*-adjust < 0.001). Although all patients in the MC group presented with GMG compared to 70.3% in the non-MC group, this difference did not reach statistical significance after adjustment (*p* = 0.003; *P*-adjust = 0.062).

In terms of inflammatory profiles, initial univariate analysis suggested potential elevations in white blood cell count (*p* = 0.046), neutrophil count (*p* = 0.044), and NLR (*p* = 0.029) in the MC group. However, after applying the Bonferroni correction, no statistically significant differences were retained for any inflammatory markers, including NLR, PLR, SII, or SIRI (all *P*-adjust > 0.05). This suggests that while inflammatory markers distinguish MG patients from healthy controls (as shown in Table 1), they may have limited utility in differentiating MC status within this specific cohort when subjected to rigorous statistical correction (Table 2).

**Table 2:** Comparison of general clinical data between the MC group and the non-MC group.

Characteristics	MC (*n* = 23)	Non-MC (*n* = 111)	*P-value*	*P-adjust*
Age (years old)	58.00 (47.00,69.00)	51.00 (39.00,67.00)	0.302	1.000
Sex (Male, %)	12, 52.2%	52, 46.8%	0.642	1.000
Hormone (%)			0.574	1.000
Y	6, 26.1%	22, 19.8%		
N	17, 73.9%	89,80.2%		
Immunosuppressor (%)			0.477	1.000
Y	4, 17.4%	12, 10.8%		
N	19, 82.6%	99, 89.2%		
Onset time (%)			0.127	1.000
EOMG	6, 26.1%	48, 43.2%		
LOMG	17, 73.9%	63, 56.8%		
Clinical classification (%)			0.003*	0.062
OMG	0, 0.0%	33, 29.7%		
GMG	23, 100.0%	78, 70.3%		
Thymoma (%)			<0.001***	<0.001***
Y	16, 69.6%	20, 18.0%		
N	7, 30.4%	91, 82.0%		
Leucocyte(10^9/L)	7.80 (5.40,11.60)	6.20 (5.00,7.85)	0.046*	1.000
Thrombocyte (10^9/L)	200 (169, 264)	215 (179.5, 256)	0.518	1.000
Lymphocyte (10^9/L)	1.20 (0.75,2.35)	1.60 (1.15,2.15)	0.127	1.000
Neutrophil (10^9/L)	5.10 (3.15,8.55)	3.80 (3.10,5.35)	0.044*	1.000
Monocyte (10^9/L)	0.30 (0.20,0.56)	0.40 (0.30,0.50)	0.943	1.000
CRP (mg/L)	5.30 (4.65,6.65)	5.30 (5.05,5.70)	0.955	1.000
HDL (mmol/L)	1.27 (1.27,1.47)	1.27 (1.13,1.47)	0.207	1.000
NLR	3.95 (1.87,9.46)	2.29 (1.66,3.80)	0.029*	0.691
MHR	0.24 (0.15, 0.45)	0.30 (0.20, 0.40)	0.759	1.000
LMR	3.83 (2.10, 4.95)	4.50 (3.50, 6.00)	0.146	1.000
PLR	166.67 (116.40, 294.44)	132.63 (106.14, 187.96)	0.165	1.000
SIRI(10^9/L)	1.18 (0.67, 3.11)	0.80 (0.52, 1.42)	0.078	1.000
SII(10^9/L)	861.9 (400.53, 1886.67)	523.96 (369.56, 869.25)	0.085	1.000

MC, myasthenic crisis; EOMG, early-onset myasthenia gravis; LOMG, late-onset myasthenia gravis; OMG, ocular myasthenia gravis; GMG, generalized myasthenia gravis; CRP, C-reactive protein; HDL, high-density lipoprotein; NLR, neutrophil-to-lymphocyte ratio; MHR, monocyte-to-HDL ratio; LMR, lymphocyte-to-monocyte ratio; PLR, platelet-to-lymphocyte ratio; SIRI, systemic inflammation response index; SII, systemic immune inflammation index. *p < 0.05, ***p < 0.001.

### Univariate logistic regression analysis of factors affecting MG onset

To identify independent risk factors for MG onset while addressing potential multicollinearity among inflammatory markers, a three-step hybrid statistical approach was employed.

First, univariate logistic regression was performed to screen candidate variables. Variables with a *p*-value< 0.2 were retained for further analysis to avoid excluding potentially relevant factors. Subsequently, Least Absolute Shrinkage and Selection Operator (LASSO) regression with 10-fold cross-validation was utilized to select the most robust predictors. The LASSO algorithm identified five key variables with non-zero coefficients: Neutrophil count, CRP, HDL, PLR, and SIRI.

**Figure 1:**
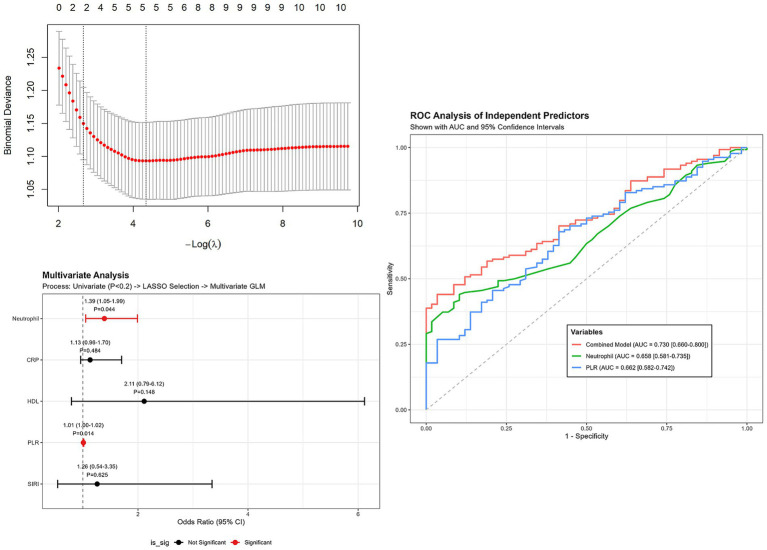
Forest plot of multivariate logistic regression analysis for risk factors associated with MG onset. The model incorporates variables selected via LASSO regression. Red dots indicate statistically significant risk factors (*p* < 0.05), while black dots indicate non-significant variables included for adjustment. OR, odds ratio; CI, confidence interval.

Finally, these LASSO-selected variables were entered into a multivariate logistic regression model. The results, visualized in the forest plot (Figure 1), demonstrated that Neutrophil count (OR: 1.39, 95% CI: 1.05–1.99, *p* = 0.044) and PLR (OR: 1.01, 95% CI: 1.00–1.02, *p* = 0.014) were significantly associated with MG onset. This indicates that even after adjusting for other inflammatory indices selected by LASSO (CRP, HDL, and SIRI, which were not statistically significant in the final model), elevated Neutrophil count and PLR remain independent risk factors for the development of MG.

The ROC curve analysis further demonstrated that the combined model achieved an AUC of 0.730 (95% CI: 0.660–0.800). Among individual predictors, PLR showed an AUC of 0.662 (95% CI: 0.582–0.742), while Neutrophil count yielded an AUC of 0.658 (95% CI: 0.581–0.735) (Figure 1).

### Multivariate logistic regression analysis of factors affecting MC onset

A parallel hierarchical analytical strategy was employed to investigate the determinants of myasthenic crisis (MC). Consistent with the analysis of MG onset, candidate variables were first screened via univariate analysis (*p* < 0.2) and subsequently refined using LASSO regression to minimize multicollinearity.

This rigorous selection process pinpointed four key variables with non-zero coefficients: Thymoma, Leukocytes, NLR, and SIRI. These features were then integrated into the final multivariate logistic regression model.

**Figure 2:**
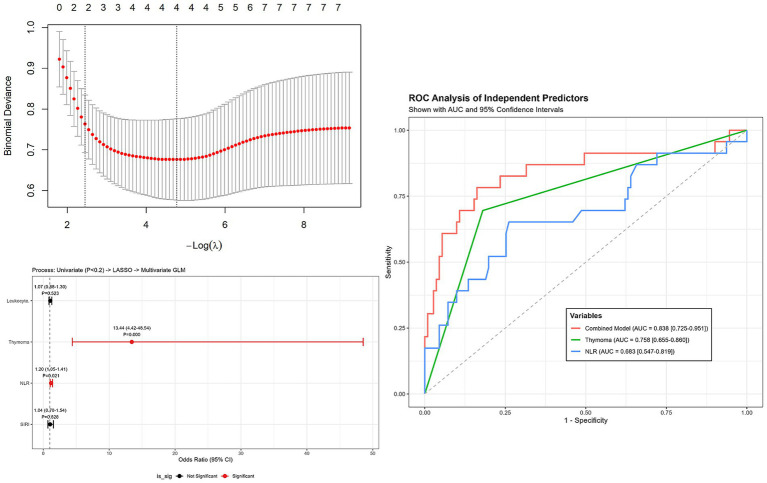
Forest plot of multivariate logistic regression analysis for risk factors associated with myasthenic crisis (MC). Variables were screened by univariate analysis (*p* < 0.2) and refined by LASSO. Thymoma and NLR were identified as independent risk factors. Error bars represent 95% confidence intervals.

As visualized in Figure 2, Thymoma demonstrated the strongest independent association with crisis events, showing a substantial odds ratio (OR: 13.44, 95% CI: 4.42–48.54, *p* < 0.001). Furthermore, NLR was confirmed as a significant independent predictor (OR: 1.20, 95% CI: 1.05–1.41, *p* = 0.021). Conversely, while White blood cell count (*p* = 0.523) and SIRI (*p* = 0.828) were included in the model for adjustment purposes, they did not exhibit statistical significance as independent risk factors.

The ROC curve analysis (Figure 2) further evaluated the predictive accuracy of these risk factors for myasthenic crisis. The multivariate model achieved a robust AUC of 0.838 (95% CI: 0.725–0.951). Individually, Thymoma yielded an AUC of 0.758 (95% CI: 0.655–0.860), while NLR demonstrated an AUC of 0.683 (95% CI: 0.547–0.819) (Figure 2).

### Comparative analysis of inflammatory markers between OMG and GMG patient groups

To investigate whether systemic inflammation correlates with the extent of muscle involvement, we compared inflammatory markers between patients with ocular MG (OMG, *n* = 33) and generalized MG (GMG, *n* = 101).

In the initial unadjusted analysis, the GMG group exhibited nominally higher levels of white blood cell count, neutrophil count, CRP, NLR, SIRI, and SII compared to the OMG group (all unadjusted *p* < 0.05). However, after strictly adjusting for multiple comparisons, only the SII remained significantly elevated in the GMG group compared to the OMG group (*P*-adjust = 0.036).

While neutrophil count and NLR showed a trend toward elevation in the GMG group, these differences were rendered marginally non-significant after adjustment (*P*-adjust = 0.063 for both). Other markers, including Leucocyte, CRP, PLR, and SIRI, showed no significant differences between the clinical subtypes in the final adjusted analysis (Table 3).

**Table 3:** Comparison of inflammatory markers between OMG and GMG groups.

Characteristics	OMG (*n* = 33)	GMG (*n* = 101)	*P-value*	*P-adjust*
Leucocyte(10^9/L)	6.10 (4.90,6.70)	6.40 (5.10,8.90)	0.044	0.396
Neutrophil (10^9/L)	3.30 (2.70,3.90)	4.40 (3.20,6.70)	0.007	0.063
CRP (mg/L)	5.10 (4.90,5.30)	5.30 (5.10,6.20)	0.018	0.162
NLR	1.88 (1.42,2.75)	2.53 (1.81,5.11)	0.007	0.063
MHR	0.32 (0.24, 0.38)	0.28 (0.17, 0.41)	0.553	1.000
LMR	4.75 (3.67, 6.20)	4.14 (3.08, 5.67)	0.205	1.000
PLR	122.94 (98.33, 160.77)	140.67 (112.5, 208.33)	0.068	0.612
SIRI	0.64 (0.49, 0.93)	0.91 (0.57, 1.75)	0.042	0.378
SII (10^9/L)	403 (332.43, 581.09)	583.33 (392.89, 1182.19)	0.004	0.036*

OMG, ocular myasthenia gravis; GMG, generalized myasthenia gravis; CRP, C-reactive protein; NLR, neutrophil-to-lymphocyte ratio; MHR, monocyte-to-HDL ratio; LMR, lymphocyte-to-monocyte ratio; PLR, platelet-to-lymphocyte ratio; SIRI, systemic inflammation response index; SII, systemic immune inflammation index. *p < 0.05.

### Comparative analysis of inflammatory markers between mild and moderate-to-severe MG groups

To elucidate the relationship between systemic inflammation and disease severity, we stratified patients according to the MGFA classification into a mild group (Class I–II, *n* = 59) and a moderate-to-severe group (Class III–V, *n* = 75).

Analysis of peripheral blood markers revealed a clear inflammatory gradient correlating with clinical severity. Even after strictly adjusting for multiple comparisons to control for Type I error, several markers remained significantly elevated in the moderate-to-severe group compared to the mild group. These included Leucocyte count (*P*-adjust = 0.027), Neutrophil count (*P*-adjust = 0.003), NLR (*P*-adjust = 0.002), and SII (*P*-adjust = 0.002).

In contrast, while PLR (*P*-adjust = 0.039) and SIRI (*P*-adjust = 0.017) initially appeared higher in severe cases, these differences were rendered non-significant following Bonferroni correction (*P*-adjust = 0.351 and *P*-adjust = 0.153, respectively). Similarly, no significant differences were observed for CRP, MHR, or LMR between the severity subgroups in the final adjusted analysis (Table 4).

**Table 4:** Comparison of inflammatory markers between mild and moderate-to-severe groups.

Characteristics	Mild (*n* = 59)	Moderate-to-Severe (*n* = 75)	*P-value*	*P-adjust*
Leucocyte(10^9/L)	6.05 (4.68, 6.70)	7.15 (5.55, 9.00)	0.003*	0.027*
Neutrophil (10^9/L)	3.40 (2.80, 4.15)	5 .00 (3.38, 7.53))	<0.001***	0.003**
CRP (mg/L)	5.20 (5.00, 5.50)	5.40 (5.00,6.30)	0.170	1.000
NLR	2.02 (1.58, 2.55)	3.45 (1.85, 6.82)	<0.001***	0.002**
MHR	0.32 (0.22, 0.39)	0.27 (0.17, 0.41)	0.369	1.000
LMR	4.67 (3.67, 6.15)	4.07 (2.98, 5.56)	0.112	1.000
PLR	128.75 (103.06, 162.19)	144.83 (111.31, 244.66)	0.039	0.351
SIRI (10^9/L)	0.73 (0.51, 1.00)	1.07 (0.62, 2.24)	0.017	0.153
SII (10^9/L)	431.72 (349.53, 580.39)	712.55 (392.24, 1466.12)	<0.001***	0.002**

CRP, C-reactive protein; HDL, high-density lipoprotein; NLR, neutrophil-to-lymphocyte ratio; MHR, monocyte-to-HDL ratio; LMR, lymphocyte-to-monocyte ratio; PLR, platelet-to-lymphocyte ratio; SIRI, systemic inflammation response index; SII, systemic immune inflammation index. *p < 0.05, **p <0.01, ***p <0.001.

## Discussion

The MG is classically defined by antibody-mediated blockade of neuromuscular transmission. However, the production of high-affinity autoantibodies is not an isolated event but the culmination of a complex systemic inflammatory cascade involving T-cell dysregulation, B-cell hyperactivation, and complement consumption (14). This study substantiates the role of systemic inflammation in MG pathophysiology, demonstrating that peripheral blood composite markers—specifically NLR, PLR, SII, and SIRI—are significantly elevated in MG patients and correlate with disease severity, clinical subtypes, and the risk of MC.

Our finding that elevated PLR is associated with MG onset suggests these cells are active participants rather than passive bystanders. Neutrophils may drive the autoimmune response through the release of Neutrophil Extracellular Traps (NETs) (15, 16). These chromatin-web structures, decorated with cytotoxic enzymes like myeloperoxidase (MPO) and citrullinated histones, can directly damage the postsynaptic membrane. More critically, NETs can act as a source of autoantigens, activating plasmacytoid dendritic cells to prime T cells against AChR, thereby sustaining the autoimmune loop (17). Similarly, the independent association of PLR with MG highlights the immunomodulatory role of platelets. Beyond hemostasis, platelets interact with leukocytes to facilitate their extravasation into inflamed tissues. While Xu et al. (6) suggested PLR predicts respiratory failure due to microthrombosis, our data indicates that platelet-lymphocyte imbalances are detectable even at disease onset. However, it is crucial to acknowledge that PLR and neutrophil counts are biologically interlinked. Since platelet activation is often secondary to systemic inflammation driven by neutrophils (18), the statistical independence of PLR observed in our model should be interpreted with caution regarding potential multicollinearity.

The distinct inflammatory profiles observed between GMG and OMG phenotypes offer clinical insights. We found that NLR, PLR, and SII were significantly higher in GMG compared to OMG. This gradient supports the hypothesis that GMG represents a state of profound systemic immune dysregulation, whereas OMG may remain an immunologically compartmentalized entity with limited systemic spillovers. The lower inflammatory burden in OMG patients aligns with their better overall prognosis and suggests that systemic biomarkers are more sensitive for monitoring patients with widespread neuromuscular involvement (19).

The MC remains the most feared complication of MG. Our study reinforces the strong link between thymoma and MC, while uniquely identifying NLR as an independent inflammatory predictor. In thymoma-associated MG, the neoplastic thymic microenvironment is characterized by the ectopic expression of muscle-like epitopes and defective negative selection, leading to the continuous export of autoreactive T cells into the periphery (20). This “thymus-blood” axis likely fuels the systemic inflammation reflected by elevated NLR. Consequently, a rising NLR in a patient with thymoma may serve as an early warning signal for impending clinical deterioration, necessitating closer respiratory monitoring.

While SII and SIRI were elevated in MG patients, their predictive value in multivariate models was less robust than NLR or PLR. It has been hypothesized that SII reflects the balance of the Th17/Treg axis, a critical immunoregulatory pathway in MG (21, 22). However, without direct flow cytometric validation in our cohort, this mechanism remains speculative. The variability of SII and SIRI in our study may also stem from the high fluctuation of platelet and monocyte counts in response to minor physiological stressors. Future studies with larger sample sizes are needed to determine if these indices offer incremental value over the simpler NLR.

Several limitations warrant consideration. First, the retrospective, single-center design introduces selection bias, particularly the predominance of hospitalized GMG cases, which may limit the generalizability of findings to mild outpatient cohorts. Second, although active infection was a strict exclusion criterion, the influence of prior corticosteroid or immunosuppressive therapy—essential for ethical disease management—could not be fully eliminated as a confounding factor. Finally, these biomarkers are non-specific indicators of systemic stress; their clinical interpretation must always be integrated with specific neurological assessments.

## Conclusion

In conclusion, peripheral blood inflammatory biomarkers provide a readily available window into the systemic immune status of MG patients. Elevated PLR and neutrophil counts are hallmarks of MG onset, potentially reflecting NETs-mediated autoimmunity. Conversely, elevated NLR, particularly in the presence of thymoma, signals a heightened risk of myasthenic crisis. Incorporating these cost-effective markers into routine practice may refine risk stratification and guide more aggressive monitoring for high-risk patients.

## Data Availability

The raw data supporting the conclusions of this article will be made available by the authors upon reasonable request.
